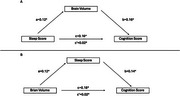# Bidirectional Mediation Effects of Sleep and Brain Volume on Cognition in Older Adults

**DOI:** 10.1002/alz70856_097781

**Published:** 2025-12-24

**Authors:** Shuo Qin, Eric Kwun Kei Ng, ChunSiong Soon, XinYu Chua, Juan Helen Zhou, Woon‐Puay Koh, Michael Wei‐Liang Chee

**Affiliations:** ^1^ National University of Singapore, Singapore, Singapore, Singapore; ^2^ Yong Loo Lin School of Medicine, National University of Singapore, Singapore, Singapore; ^3^ National University of Singapore, Singapore, Sinagpore, Singapore

## Abstract

**Background:**

Sleep fragmentation and irregularity have been associated with reduced performance in tasks evaluating memory, executive functions, and processing speed. In older adults, lowered performance in these cognitive domains is associated with decreased brain volumes, especially cortical gray matter and hippocampal volumes. However, the mechanisms by which sleep and brain volumes interact to influence cognition remain poorly understood. This study explored the bidirectional mediation effects of sleep and brain volume on cognitive performance in community‐dwelling older adults.

**Method:**

The current study is part of the ongoing SG70 study. Eight hundred community‐dwelling older adults wore an Oura ring to track habitual sleep patterns for 1 month and completed a comprehensive neurocognitive battery. Using partial least squares correlation (PLSC) analysis, we identified a profile of high sleep regularity and low fragmentation linked to better cognitive performance, explaining 68% of the covariance between sleep and cognition. Sleep and cognition scores from the significant PLSC component were extracted for 467 SG70 participants eligible for MRI. Cortical gray matter and bilateral hippocampal volumes from these participants were extracted using Freesurfer. A composite score representing overall gray matter volume was computed using confirmatory factor analysis. Two mediation analyses adjusted for age, gender, and estimated intracranial volume were conducted to examine the bidirectional relationship between sleep scores and brain volumes and their influence on cognition.

**Result:**

Correlation analyses revealed significant associations between brain volume composite scores and both sleep (*r* = 0.18) and cognition (*r* = 0.15) scores from the significant PLSC component. In the first mediation analysis, brain volume partially mediated the relationship between sleep and cognition (Figure 1a). In the second analysis, sleep partially mediated the relationship between brain volume and cognition (Figure 1b). These results demonstrate a bidirectional mediation effect, where brain volume and sleep mutually influence the relationship between sleep, brain health, and cognition.

**Conclusion:**

The bidirectional albeit small mediation effect suggests that gray matter atrophy and irregular/fragmented sleep influence each other to negatively affect cognition in community‐dwelling older adults.